# Genetic association study identifies a functional CNV in the *WWOX* gene contributes to the risk of intracranial aneurysms

**DOI:** 10.18632/oncotarget.7546

**Published:** 2016-02-21

**Authors:** Jin Fan, Wen Sun, Min Lin, Ke Yu, Jian Wang, Dan Duan, Bo Zheng, Zhenghui Yang, Qingsong Wang

**Affiliations:** ^1^ Department of Neurology, Chengdu Military General Hospital, Chengdu, China; ^2^ Department of Neurology, Jinling Hospital of Nanjing University, Nanjing, China; ^3^ Department of Neurology, Fuzhou General Hospital of Nanjing Command, PLA and Clinical Medical College of Fujian Medical University, Fuzhou, China

**Keywords:** intracranial aneurysm, gene, SNP, risk factor, WWOX

## Abstract

Intracranial aneurysms (IAs) accounts for 85% of hemorrhagic stroke. Genetic factors have been known to play an important role in the development of IAs. A functional CNV (CNV-67048) of human WW domain-containing oxidoreductase (*WWOX*), which has been identified as a tumor suppressor gene in multiple cancers, was identified to be associated with gliomas risk previously. Here, we hypothesized that the CNV-67048 could also affect susceptibility of IAs. Based on a two-stage, case^−^ control study with a total of 976 patients of IAs and 1,200 matched healthy controls, we found the effect size for per copy deletion was 1.35 (95% CI = 1.16–1.57; *P*_trend_ = 1.18 × 10^−4^). Compared with the individuals having no deletion, significantly higher risk of IAs was detected for both subjects carrying 1 copy deletion (OR = 1.24, 95% CI = 1.02–1.52) and subjects carrying 2 copy deletion (OR = 1.77, 95% CI = 1.24–2.53). Real-time PCR was used to confirm the abnormal expression of *WWOX* in tissues of IA patients and influence of genotypes of CNV-67048. The expression level of *WWOX* in IA tissues was significantly lower than that in corresponding normal tissues (*P* = 0.004), and the deletion genotypes of CNV-67048 have lower *WWOX* mRNA levels in both tumor tissues and border tissues (*P* < 0.01). Our data suggests that the deletion genotypes of CNV-67048 in WWOX predispose their carriers to IAs, which might be a genetic biomarker to predict risk of IAs in Chinese.

## INTRODUCTION

Intracranial aneurysms (IAs) account for about 80–85% of non-traumatic subarachnoid hemorrhages (SAH) [1–3]. The prevalence of IAs is about 1–5% (10 million to 12 million persons in the United States) and the incidence is 1 per 10,000 persons per year in the United States (approximately 27,000), with incidence highest in 30- to 60-year-olds [4, 5]. Women own more cases of IAs, by a ratio of 3 to 2 [4]. Little is known about the molecular pathogenesis of IAs. They are thought to result from the interplay of environmental and genetic factors [6]. Previous studies have indicated that hypertension, hypercholesterolemia, cigarette smoking and female gender are risk factors for IAs [7]. Also, there is increasing evidence to suggest that genetic factors play an important part in the pathogenesis of IAs [6, 8, 9].

Many studies have revealed that copy number variations (CNVs) contributed to part of the missing heritability for complex traits after the GWAS era [10–20]. Human WW domain-containing oxidoreductase (*WWOX*) is a tumor suppressor that has been reported to lose function due to genetic alterations carcinogenesis [21, 22]. It is found in all eukaryotes and play an important role in the regulation of a wide variety of cellular functions such as protein degradation, transcription, and RNA splicing [23–25]. The human *WWOX* gene (OMIM: 605131) is located on chromosome 16q23.3–24.1, a region spanning over the common fragile site 16D (FRA16D). Somatic and germline mutations of *WWOX*, including loss of heterozygosis (LOH), homozygous deletions and chromosomal translocations, has been reported to evolve in carcinogenesis and development in several types of cancers [22]. Very interestingly, we previously identified that deletion genotypes of CNV-67048 in *WWOX* gene predispose their carriers to gliomas, which are the most common primary tumors of the central nervous system (CNS) and representing more than 80% of all malignant brain tumors [21]. Basing on the evidence above, we hypothesized that the CNVs in *WWOX* might be also associated with risk of IAs by disturbing the function of *WWOX*. To test this hypothesis, we performed a two-stage, case-control study among Chinese people to evaluate whether *WWOX* CNV-67048, a representative CNV in the *WWOX* gene region, contributes to the risk of IAs.

## RESULTS

Totally included in this study were 976 cases of IAs and 1200 matched healthy controls. As shown in Table 1, all the demographic characteristics of patients with IAs and controls are summarized. There were no statistically significant differences between groups with respect to age, gender, smoking status, drinking status, and body mass index (BMI) in both discovery stage and validation stage (all *P* value > 0.05). However, the hypertension rates of patients with IAs were significantly higher than those of healthy controls in both discovery stage (*P* < 0.001) and validation stage (*P* < 0.001).

**Table 1:** Characteristics of IAs patients and healthy controls used in this study

Category	Discovery stage			Validation stage		
	Cases (*N* = 400)	Controls (*N* = 600)	*P* Value	Cases (*N* = 576)	Controls (*N* = 600)	*P* Value
Age (yr)						
Mean ± SD	54.0 ± 6.7	53.8 ± 7.1	0.655	53.2 ± 4.1	53.7 ± 6.3	0.108
Gerder						
Male	167 (41.8%)	249 (41.5%)	0.937	228 (39.5%)	233 (38.8%)	0.792
Female	233 (58.2%)	351 (58.5%)		348 (60.5%)	367 (61.2%)	
Hypertension						
Yes	240 (60.0%)	127 (21.2%)	***P* < 0.001**	337 (58.5%)	120 (20.0%)	***P* < 0.001**
No	160 (40.0%)	473 (78.8%)		239 (41.5%)	480 (80.0%)	
Ever smoker						
Yes	74 (18.5%)	86 (14.3%)	0.078	108 (18.8%)	88 (14.7%)	0.060
No	326 (81.5%)	514 (85.7%)		468 (81.2%)	512 (85.3%)	
Ever drinker						
Yes	89 (22.3%)	121 (20.1%)	0.428	138 (23.9%)	123 (20.5%)	0.154
No	311 (77.7%)	479 (79.9%)		438 (76.1%)	477 (79.5%)	
Body mass index (kg/m^2^)	24.2 ± 3.6	23.9 ± 3.1	0.160	24.3 ± 4.1	23.9 ± 3.2	0.061

Table 2 presented the genotype distribution of *WWOX* CNV-67048 among both patients with IAs and healthy controls in the discovery stage. Consistent with results in DGV databases, we detected three kinds of genotypes for *WWOX* CNV-67048 (no deletion, one copy deletion, and two copy deletion) in all samples. When analyzing the data using a log-additive model and adjusted for age, gender, smoking status, and Hypertension, we found that there was a significantly higher risk of IAs for per copy deletion (OR = 1.43, 95% CI = 1.14–1.80; *P*_trend_ = 1.79 × 10^−3^). Compared with the individuals having no deletion, those carrying 1 copy deletion harbored a 1.37-fold increased risk of lung cancer (OR = 1.37, 95% CI = 1.02–1.83), while those with 2 copy deletion had a much higher risk (OR = 1.88, 95% CI = 1.10–3.21).

**Table 2:** *WWOX* gene deletion and risk of IAs in discovery stage

Genotypes	No. of cases	No. of controls	OR (95% CI)^a^
no deletion	255 (63.8%)	431 (71.8%)	1.00 (reference)
1 copy deletion	115 (28.8%)	142 (23.7%)	1.37 (1.02–1.83)
2 copy deletion	30 (7.4%)	27 (4.5%)	1.88 (1.10–3.21)
per copy deletion			1.43 (1.14–1.80)
P_trend_			**1.79 × 10**^−3^

a Adjusted for age, gender, smoking status, and Hypertension.

Furthermore, the results were validated in an independent validation dataset (Table 3). A 1.29-fold increased risk of IAs were detected for per copy deletion (OR = 1.29, 95% CI = 1.05-1.58; *P*_trend_ = 0.017). When pooled together, significantly higher risk of IAs was detected for both subjects carrying 1 copy deletion (OR = 1.24, 95% CI = 1.02^−^1.52) and subjects carrying 2 copy deletion (OR = 1.77, 95% CI = 1.24^−^2.53), compared with the individuals having no deletion. The effect size for per copy deletion was 1.35 (95% CI = 1.16^−^1.57; *P*_trend_ = 1.18 × 10^−4^). Multiplicative interactions between CNV and demographic variables were evaluated using the likelihood ratio test when interaction terms were added to logistic regression models along with the main effect terms. However, we did not find any significant interaction terms on risk of IAs (data not shown).

**Table 3:** *WWOX* gene deletion and risk of IAs in validation stage and the pooled results

Genotypes	No. of cases	No. of controls	OR (95% CI)^a^
**Validation stage**			
no deletion	384 (66.7%)	429 (71.5%)	1.00 (reference)
1 copy deletion	147 (25.5%)	141 (23.5%)	1.16 (0.89–1.52)
2 copy deletion	45 (7.8%)	30 (5.0%)	1.67 (1.04–2.70)
per copy deletion			1.29 (1.05–1.58)
P_trend_			**0.017**
**Pooled results**			
no deletion	639 (65.5%)	860 (71.7%)	1.00 (reference)
1 copy deletion	262 (26.8%)	283 (23.6%)	1.24 (1.02–1.52)
2 copy deletion	75 (7.7%)	57 (4.7%)	1.77 (1.24–2.53)
per copy deletion			1.35 (1.16–1.57)
P _trend_			**1.18 × 10^−4^**

a Adjusted for age, gender, smoking status, and Hypertension.

**Figure 1:**
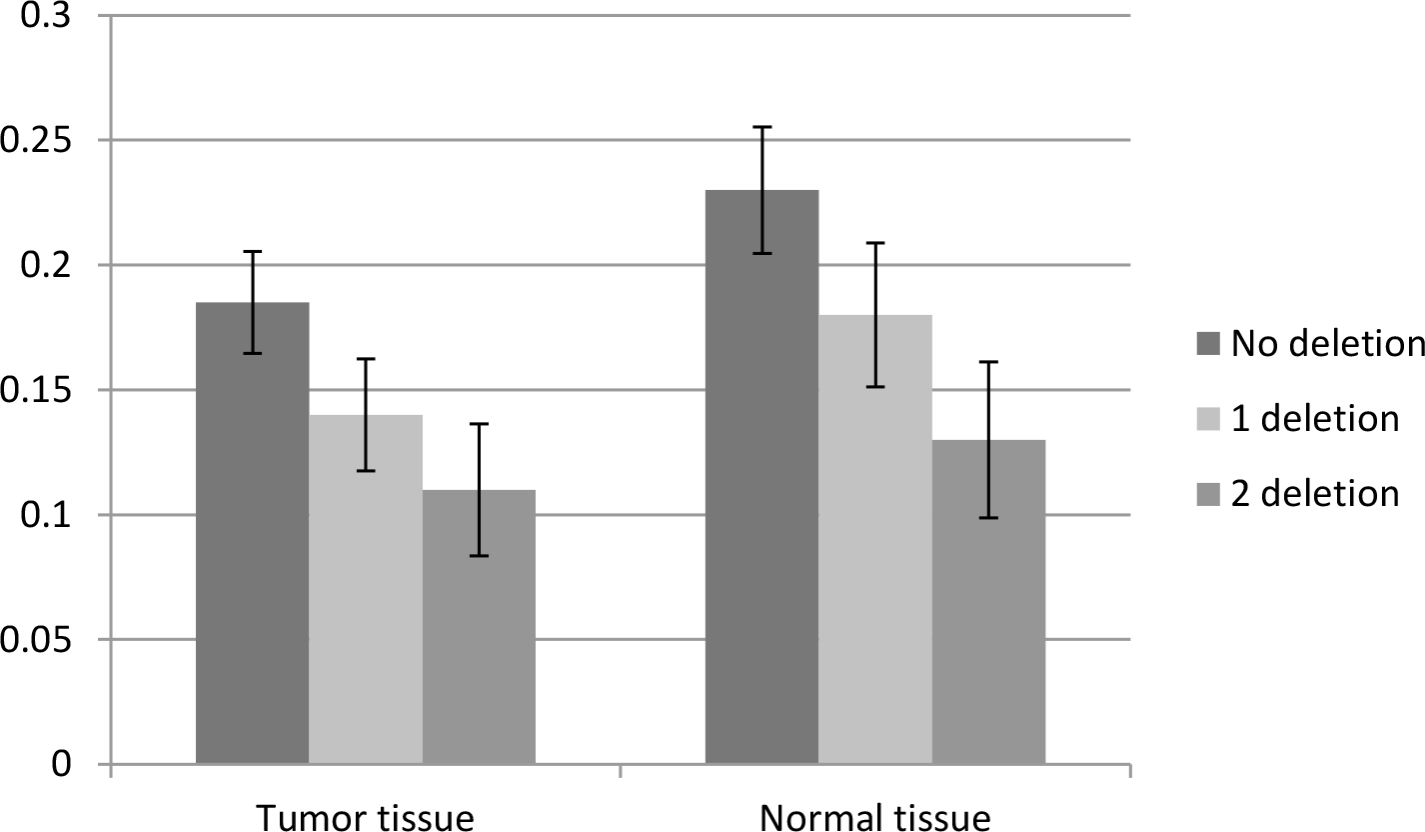
Association between the CNV-67048 and *WWOX* expression. The relative mRNA levels of WWOX in IA tissues compared with border normal tissues with different genotypes

**Figure 2:**
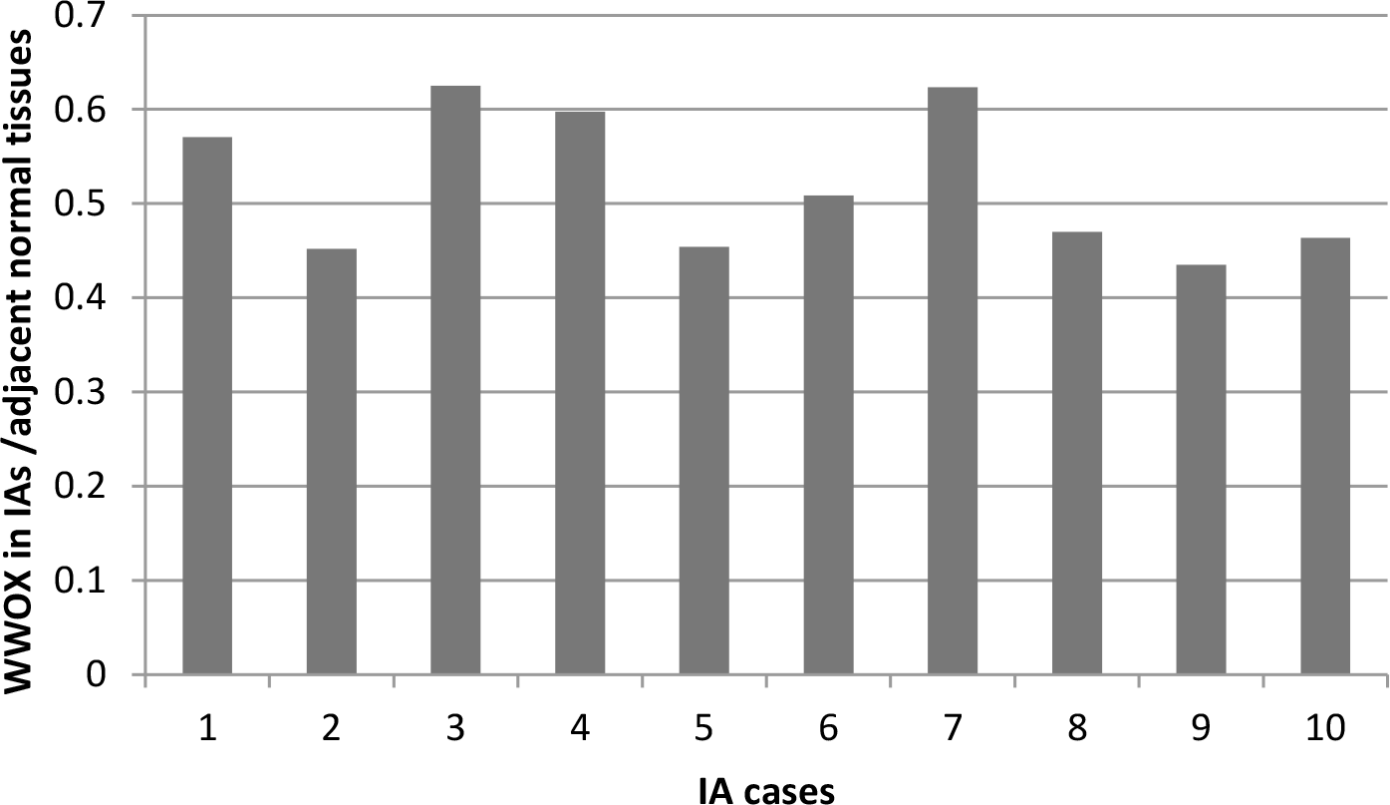
Densitometry measurements from Western blots of WWOX in paired IA tissues and adjacent normal tissues (WWOX in IAs tissues/adjacent normal tissues)

To confirm the abnormal expression of *WWOX* in tissues of IA patients and influence of genotypes of CNV-67048, we evaluated the *WWOX* levels in 70 paired tissues of IA patients and corresponding normal tissues. As shown in Figure 1, the expression level of *WWOX* in IA tissues was significantly lower than that in corresponding normal tissues (*P* = 0.004). The deletion genotypes of CNV-67048 have lower *WWOX* mRNA levels in both tumor tissues (*P* = 0.001) and border tissues (*P* = 0.002). Then, to determine whether WWOX protein expression was altered in IAs, we also performed Western blot analysis of lysates from 10 paired IA tissues and adjacent normal tissues. As shown in Figure 2, densitometry measurements from Western blots revealed that all of the IA tissues had an average of 48% reduction of WWOX levels compared to adjacent normal tissues when normalized to actin.

## DISCUSSION

Fully exploration of CNVs and their role in carcinogenesis could possess a large number of potential clues for developing novel therapeutic agents for IAs. In the current two-stage, case–control study, we found that the CNV-67048 in *WWOX* was significantly associated with increased risk of IAs. The deletion genotypes of CNV-67048 were related with a higher rate of IAs and lower expression of *WWOX* than the no deletion genotype in IA tissues. To the best of our knowledge, this is the first study conducted to reveal a functional CNV in *WWOX* predisposing development of IAs. This provides evidence to implicate *WWOX* gene deletion as a novel susceptibility factor for IAs.

Rupture of IAs causes subarachnoid hemorrhage (SAH), a serious subtype of stroke, which leads to fatality in more than 50% of the cases and results in significant disability in 30% of the cases [26, 27]. Screening the susceptibility loci is an important work to effectively prevent the hazards of IAs. Although 9 genome-wide association studies (GWAS) have been conducted since 2008, only one locus (9p21.3) was consistently replicated across different studies, which means the majority of genetic factors for IAs needs further exploration [6]. Furthermore, the number of reports examining susceptibility loci for IA among Asian populations is particularly limited.

In 2000, Bednarek et al. [28] first identified that *WWOX*, a novel WW domain-containing protein mapping to human chromosome 16q23.3-24.1, as a region frequently affected in breast cancer. The ORF of *WWOX* is 1245 bp long, encoding a 414-amino acid protein with nine exons [29]. The distinct methylation patterns of *WWOX* could also influence the disease status of lung squamous cell carcinomas, invasive breast carcinomas, normal mammary tissues, and bladder transitional cell carcinomas [30]. Nowakowska et al. [31] found that *WWOX* expression were related with cell cycle and apoptosis regulation in neuroblastoma. In the current study, we showed that *WWOX* expression was lower in IA tissues compared with their adjacent normal tissues. Germline CNVs is a potential induction factor of somatic genetic alterations, which may be involved in the carcinogenesis process and development [32–35]. *WWOX* CNV-67048, which maps to chr16:76,929,120-76,942,453, has been linked to risk of lung cancer, gliomas, and COPD recently [21, 22, 36]. In our study, we first find that the *WWOX* gene deletion (CNV-67048) was associated with increased risk of IAs. As known, CNVs can directly influence gene expression and phenotypic variation, disrupt gene structure, alter gene dosage and indirectly regulate gene function through position effects. In this study, the deletion genotypes of CNV-67048 have lower *WWOX* mRNA levels in both tumor tissues and their borderline normal tissues, compared with the no deletion genotype. Findings of our study fit with current research about the suppressor gene function of *WWOX*.

The pleotropic roles of WWOX was not only restricted to tumor suppression, but also expanded to neuropathy as well as metabolic diseases. Dayan et al. [37] reported that altering metabolism from glycolysis to oxidative phosphorylation causes stable increase in steady-state levels of transcripts of the WWOX gene. Additionally, exposure to hypoxic conditions could cause a down-regulation of WWOX mRNA. Due to WWOX often exhibits homozygous deletions and translocation breakpoints under multiple cellular stresses induced by extrinsic or intrinsic factors, such as hypoxia, UV, and DNA damage regents, evidence has shown that WWOX that contains a short-chain dehydrogenase/reductase (SDR) domain is involved in steroid metabolism and bone development, while reduced or lost expression of WWOX will lead to development of metabolic disease [38]. Aldaz et al. [39] also reviewed that WWOX acted at the crossroads of cancer, metabolic syndrome related traits and CNS pathologies. Functionally, the WW domain is not only a tumor suppressor, but also a participant in molecular interactions, signaling, and apoptosis in many diseases. Studies also revealed the potential mechanism by which WWOX/WOX1 may participate in the pathogenesis of AD with a focus on cell death signaling pathways in neurons [40].

Strengths of current study includes: (1) our large sample size and a two-stage, case– control study design; (2) functional analyses further supported the findings that the CNV has significant association with IA risk. However, some limitations should also be considered: (1) although the sample size was large enough for the main analyses, the statistical power for the interaction analyses was still limited; (2) the potential bias for case-control study; (3) the extrapolation of our results to other ethnic groups.

In conclusion, this study found that the deletion genotypes of CNV-67048 in *WWOX* was significantly associated with an increased risk of IAs among Chinese population, and reduced the expression of *WWOX in vivo*. The results suggest that the CNV-67048 in *WWOX* gene may be a new biomarker for susceptibility of IAs. Validations with larger population-based studies in different ethnic groups and further research into the function of *WWOX* deletion may be warranted.

## MATERIALS AND METHODS

### Subjects

Subjects included in this study were geographically homogenous Han Chinese derived from Chengdu Military General Hospital, Jinling Hospital of Nanjing University, and Fuzhou General Hospital of Nanjing Command before June 2015. Ruptured IAs were diagnosed according to the suggestive symptoms of SAH, subarachnoid blood on CT, or a proven aneurysm at angiography (angiogram, CT, or MRA/or MRI) or at surgery; un-ruptured IAs were ascertained using CT, MRA (or MRI), angiography, surgery, or autopsy. All suspected IAs were confirmed by digital subtraction angiography (DSA). Health controls were matched for area of residence, age and gender to eliminate the effect of population stratification by heterogeneity. A structured questionnaire was used to elicit detailed information on demographic factors. Then 5 ml blood samples were obtained from the subjects who participated in the study. Finally, included in this study were 400 subjects diagnosed with IAs, and 600 healthy controls in the discovery stage, as well as 576 IAs patients and 600 healthy controls in an independent validation stage. The study complied with the Declaration of Helsinki and was approved by the appropriate Institutional Review Board and Ethics Committee, and all participants provided written informed consent.

### CNV selection and genotyping

The procedure of CNV selection and genotype has been described previously [21]. Briefly, through searching the DGV database, we found only two deletion of copy number CNVs (CNV-38092 and CNV-67048) to exist in Chinese populations with the minor allele frequency (MAF) > 0.05. As reported previously [21], 98% of individuals shared the deletion of CNV-38092 and CNV-67048, which means they could be used as the proxy of each other. In current study, we finally selected CNV-67048 as the representative CNV. Real-time qualitative PCR (qPCR) were used to conduct copy number analyses, and all CNV calls were conducted by two independent staff members.

### *WWOX* expression detection and western blot

The mRNA levels of *WWOX* were detected by SYBR-Green real-time PCR in IAs tissues and border of the IAs tissue of 70 cases. The primers were designed as previous [21, 22]: 5′-TGG GTT TAC TAC GCC AAT C-3′(forward) and 5′- GTC CGTTCT CAT CAG TTT CT -3′(reverse) to amplify 124 bp cDNA sequences from exon 2 to exon 3 of *WWOX*, while the b-actin was used as an internal reference gene. Western blot analyses were performed using protein extracts obtained from 10 paired IA tissues and adjacent normal tissues. 50 μg of total protein was separated by 12.5% SDS–PAGE and transferred onto PVDF membranes (Millipore, Billerica, MA, USA). Immunodetection was performed using Protein Detector™ (KPL, Gaithersburg, MD, USA) Western blotting reagents as described by the manufacturer. Actin was detected using monoclonal anti-actin antibody (1:1000) and HRP conjugated anti-mouse secondary antibody (1:5000). Quantitation of western blot autoradiographs was done using a Kodak digital science Image Station 440CF.

### Statistical analyses

Cases and controls were compared with the Chi-square or *T* test for categorical or continuous variables, respectively. Unconditional logistic regression models were used to evaluated the associations between the CNV-67048 and risk of IAs using odds ratios (ORs) and 95% confidence intervals (CIs). ORs were estimated for 1 copy and 2 copy deletion genotypes compared with no deletion genotype. The OR (95% CI) was also estimated for per copy deletion based on a log-additive model and adjusted for age, gender, smoking status, and Hypertension. One-way ANOVA tests were used for analyzing the association between CNV-67048 genotypes and *WWOX* expression. All statistical analyses were conducted with SAS version 9.2 (SAS Institute Inc.). All statistical tests were 2-tailed, and *P* < 0.05 was interpreted as statistically significant.
